# Clinical application of the C2 pars screw technique in the treatment of ossification of the posterior longitudinal ligament

**DOI:** 10.1186/s12891-022-05136-9

**Published:** 2022-02-24

**Authors:** Zheng Wang, Heng-Rui Chang, Zhen Liu, Zhi-Wei Wang, Wen-Yuan Ding, Da-Long Yang

**Affiliations:** Department of Spinal Surgery, The Third Hospital of Hebei Medical University, 139 Ziqiang Road, Shijiazhuang, 050051 PR China

## Abstract

**Background:**

Our research was designed to decide whether the application of C2 pars screws is an alternative choice for patients with OPLL involving the C2 segment.

**Methods:**

A total of 40 patients who underwent cervical laminectomy with fusion (LF) from C2 to C6 for OPLL were reviewed. Among them, C2 pedicle screws were placed in 23 patients, who were the pedicle group, and C2 pars screws were placed in 17 patients, who were the pars group. The screw placement and vertebral artery (VA) anatomy presented by standard CT. General clinical characteristics and health-related outcomes were evaluated and compared preoperatively and during the follow-up period.

**Results:**

The Pars group tended to have a shorter operation duration and less blood loss than the pedicle group (operation time: 115.29 ± 28.75 vs 133.48 ± 26.22, *p* = 0.044; blood loss: 383.53 ± 116.19 vs 457.83 ± 145.45, *p* = 0.039). Operation time and blood loss were both independently related to the pars group (operation time: OR = 0.966, *p* = 0.021; blood loss: OR = 0.993, *p* = 0.046). The idealization and acceptability of C2 screws in the pars group exceeded those in the pedicle group (100% vs 91.3%). However, no statistically obvious variation in the included complications or health-related outcomes between the pedicle and pars groups was observed.

**Conclusion:**

In the treatment of patients with OPLL involving the C2 segment, the application of C2 pars screws is an alternative choice, which is not only safer but also reduces the amount of blood loss, shortens the operation time and obtains a more ideal screw placement.

## Introduction

As a multifactorial disease caused by ectopic bone hyperplasia and posterior longitudinal ligament calcification [1], Ossification of the posterior longitudinal ligament (OPLL) is the most common among males, the elderly and Asian patients [2] and can easily lead to spinal cord injury after minor trauma. Both anterior and posterior approaches can realize effective decompression and relieve clinical neurological symptoms. In particular, when OPLL involves the C2 segment, its complicated anatomy makes anterior decompression surgery difficult [3]. Posterior surgery is considered to be a commonly used surgical method, including laminoplasty, laminectomy and laminectomy with fusion. C2 fixation is very important in the treatment of many occipital cervical, atlantoaxial and subaxial spinal diseases. It provides one of the strongest fixation points for the cervical spine. Nevertheless, the unique and greatly varying C2 anatomy makes this level of instrument challenging and prone to potentially serious and even life-threatening complications including vertebral artery (VA) injury, spinal cord injury or nerve injury. Recently, advances in instrumental technology have helped treat cervical spine lesions by offering critical improvements in structural stability and strength and more choices in screw placement. The development of the C2 posterior instrumental method enables surgeons to more closely select the stabilization approach of their choice based on the anatomy of a specific patient [4, 5], and increasingly diverse screw placement methods have added increasing versatility to the treatment of cervical diseases. Both C2 pedicle screws and C2 pars screws (sometimes called isthmic screws), as good fixation approaches in posterior upper cervical spine surgery [6, 7], are receiving increasing attention. Compared with C2 lateral mass screws, C2 pedicle screws and C2 pars screws have better bone grip [8, 9]. In particular, C2 pars screws have become prevalent as an approach to decrease the risk of screw-related complications (such as VA injury) during the perioperative period [10]. This technique of C2 screw placement is obviously a viable option for posterior C2 instruments, and it may reduce the risk of VA injury, especially among patients with challenging VA anatomy. The C2 pars screw uses a smooth nonthreaded part that protrudes above the bone surface of the side block to minimize stimulation of the C2 nerve root and facilitate connection with the rod. Especially in the case of a high-riding VA or medially positioned VA, using 16 mm pars screws (not reaching the pedicle) or laminar screws is a better choice than pedicle screws. However, compared with C2 pedicle screws, it remains controversial as to whether pars screws provide enough rigid fixation from the perspective of biomechanics [11] and whether they are accompanied by a higher incidence of false joints from the perspective of clinical efficacy [12–14]. Moreover, these descriptions of screw placement are all for upper cervical surgery. There has never been a report about whether pedicle screws can be placed when OPLL extends to the C2 segment. Therefore, the purpose of our research was to explore whether the application of C2 pars screws is an alternative for patients with OPLL involving the C2 segment.

## Methods

### Patient population

**Fig. 1:**
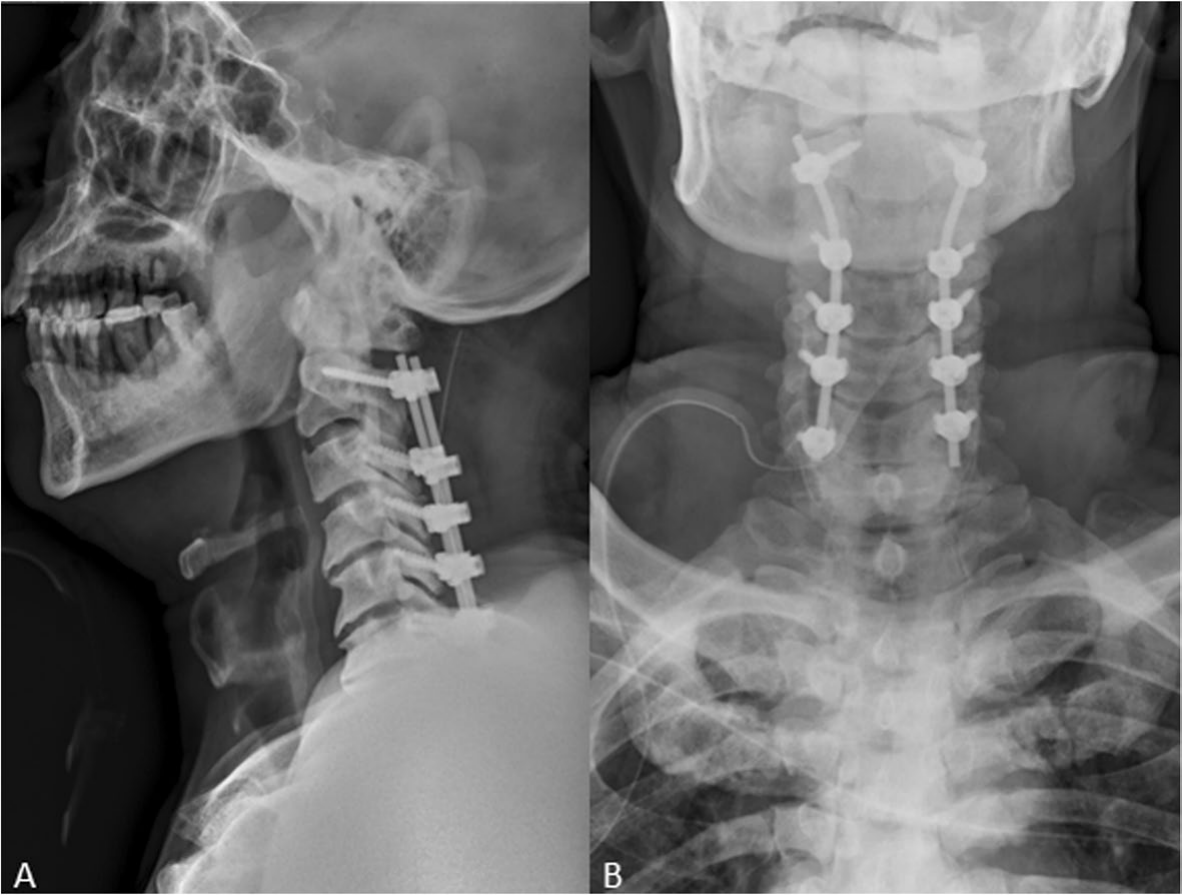
**A** Lateral and **B** anterior X-rays show that pedicle screws were inserted into C2

**Fig. 2:**
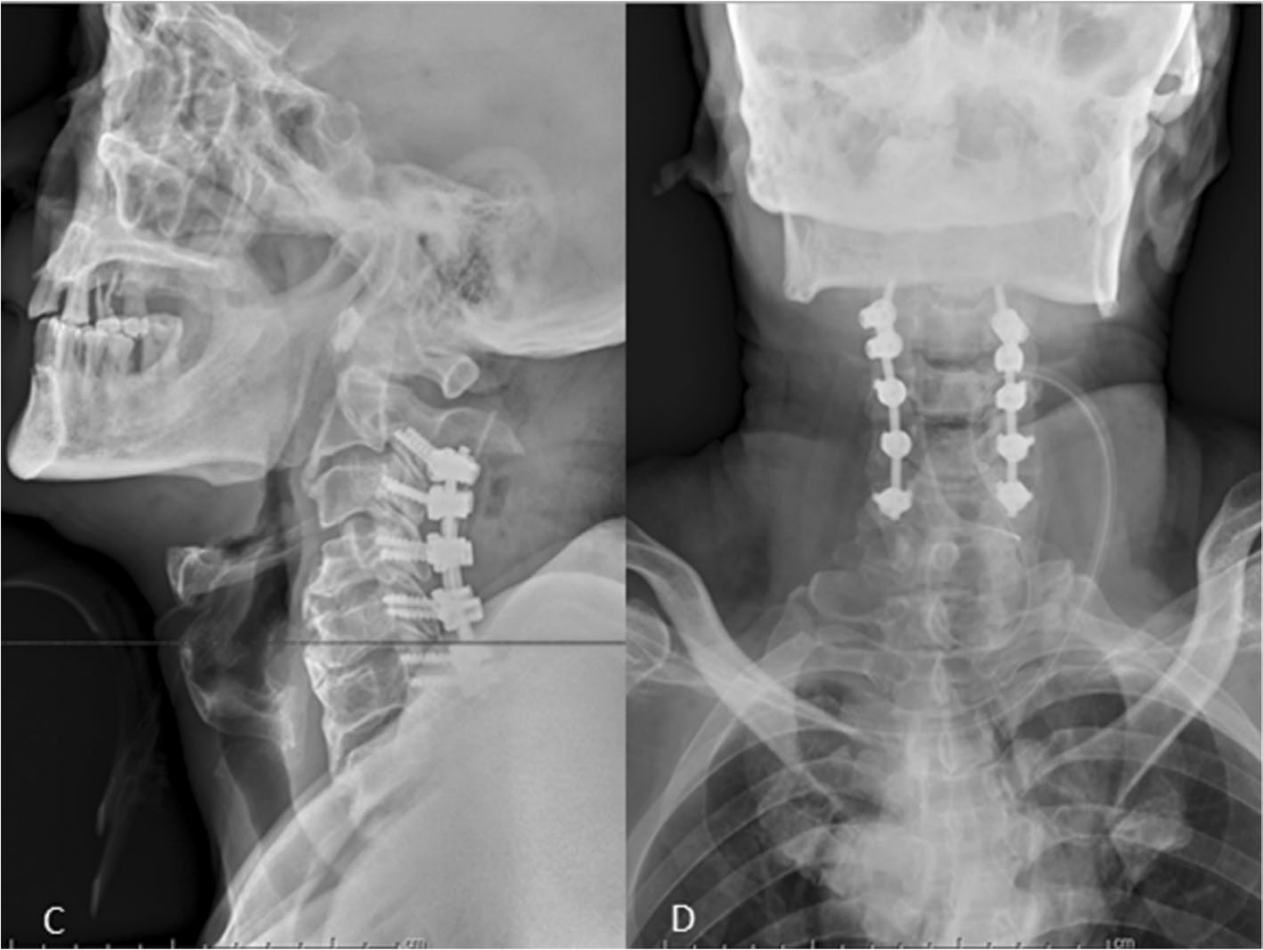
**C** Lateral and **D** anterior X-rays show that pars screws were inserted into C2

A total of 40 patients who received cervical laminectomy with fusion (LF) for OPLL between January 2010 and January 2017 were reviewed retrospectively. Among them, C2 pedicle screws were placed in 23 patients, and C2 pars screws were placed in 17 patients. All the surgeries were finished successfully (Figs. 1 and 2). Patients who: (1) were diagnosed OPLL involving C2 to C6 by computed tomography (CT); (2) completed radiographic and clinical data; and (3) were seen for follow-up 24 months after surgery were excluded. We excluded patients with: (1) previous surgery of the cervical spine; (2) cervical fractures, tumors, and metabolic disorders; (3) a follow-up period of less than 2 years; and (4) radiological data too unclear to collect.

All operations were carried out by the same experienced orthopedic surgeon. The decision on which screw placement method to use to fix C2 is based on the surgeon’s preference and the screw placement and VA anatomy presented by standard preoperative CT. C2 pars screws were applied in patients with abnormal anatomy of pedicle or with high-riding VA. Intraoperative O-arm fluoroscopy and bony anatomical landmarks are mainly applied to guide the placement of screws. No other forms of navigation are used. Electronic medical records to assess general perioperative complications, with a focus on the identification of the complications of screw misalignment including VA injury, spinal cord injury, neurologic injury and cerebrospinal fluid (CSF) leakage provided clinical data. Clinical and neurological observations of each patient’s postoperative process were performed to ensure that there was no evidence of nerve death or stroke due to VA injury before discharge. Radiological analysis includes evaluating cervical CT scans for the quantification of the patient’s bone and vascular anatomy, the classification of the accuracy of C2 screw placement and the judgement of screw looseness and failure.

The classification of screw positioning was made based on the modification below of the All India Institute of Medical Sciences result-based classification described for grading pedicle screws [15]: Type I: Ideal placement-screw threads are fully within the bony cortex. Type II: Acceptable placement less than 50% of the diameter of the screw acts against the surrounding cortex, and less than 1 mm of protrusion from the anterior cortex for transarticular and pedicle screws. Type III: Unacceptable placement-clear violation of the transverse foramen or spinal canal, despite of clinical neurovascular complications.

### Operative procedure

**Fig. 3:**
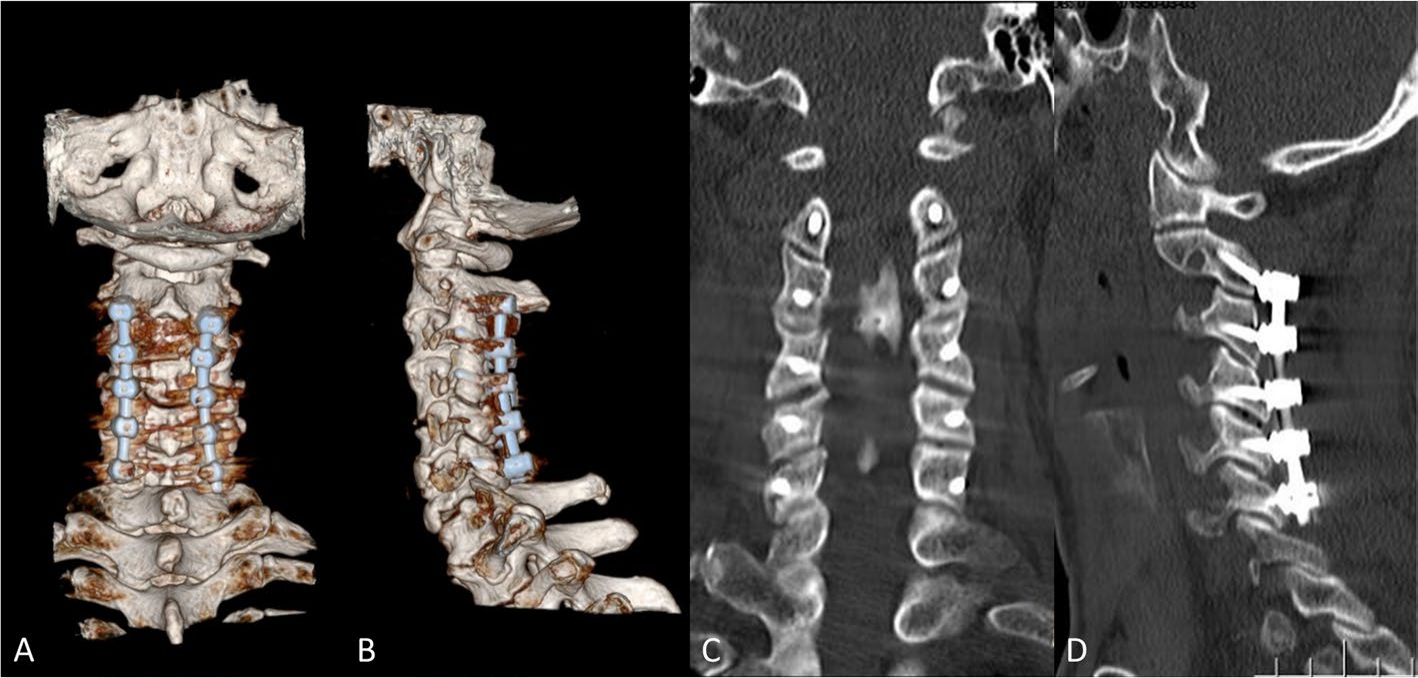
Three-dimensional surface renderings show typical trajectories of C2 pars screws from the dorsal (**A**) and right side (**B**) perspectives. CT scans show typical trajectories of C2 pars screws from the dorsal (**C**) and right side (**D**) perspectives

The patients were placed in the standard prone position under general anesthesia. LF was performed by applying a posterior screw-rod system for OPLL patients with straight, lordotic cervical curvature, or segmental instability to stop local kyphosis after decompression. The screw placements of C2 were as follows:

- C2 pedicle screw: The reference frame was connected to the Mayfield clamp or spinous process after exposure. The intraoperative CT images were got by O-arm navigation (Medtronic, PLC, Dublin, Republic of Ireland). Then, the transfer of images to the operational guidance system (Medtronic, PLC) of the stealth station was made, and the registration and verification of instruments were made for accuracy. To optimize the screw length and avoid neurovascular structure, the entry point of the pedicle screw was determined by CT image guidance. Pedicle screw placement entered the medial and upper quadrants of the pars surface. When the image guidance system is used, the navigation drill and power drill advance along the required trajectory. Then, the ball-tip probe was applied for the exploration of whether there was any breach. The screws were placed on the same track after tapping.
- C2 pars screw: After the C2 lamina and lateral mass were exposed, C2 pars were dissected directly under the periosteum. Then, a pilot hole was drilled with a high-speed drill. The entry point of the pars screw is usually inferior and lateral to that of the pedicle screw, about 4 mm lateral and cranial to the inferomedial edge of the C2–3 facet joint. Under direct anatomical visualization, the screw was inserted freehand, and the trajectory pointed to the junction of the pars and superior facet joint (Fig. 3A-D).

We applied a pars screw or pedicle screw at C2 and a lateral mass screw at C3–6 after achieving complete decompression. Rod instrumentation was then performed and cut into a suitable length to fit each patient and bent to fit the lordotic curve during fixation. All patients in both groups were told to wear a Philadelphia collar for 2–4 weeks and perform moderate physical function exercises.

### Health-related outcomes

Health-related outcomes were assessed during the preoperative and follow-up periods, including the Japanese Orthopaedic Association (JOA) (score 0–17) and recovery rate (RR) (postoperative score-preoperative mark)/(17-preoperative mark) × 100%.). An RR higher than 75% was regarded excellent, 50 to 75% good, 25 to 49% fair, and less than 25% poor. Axial pain around the posterior neck or suprascapular areas was assessed by visual Analog Scale (VAS) scoring system (range 1–10) and Neck Disability Index (NDI) (range 0–50).

### Statistical analysis

Data are presented as the number of subjects in each group or mean ± standard deviation and were explored with the SPSS program (version 22.0; SPSS Inc., Chicago, IL, USA). Each independent variable was compared between the two groups using the independent-sample t-test or Mann-Whitney U test for continuous variables and the χ2 test, continuity correction test or Fisher’s exact test for categorical variables. Each continuous variable was compared preoperatively and at follow-up using the Wilcoxon signed rank test. Relevant factors of pars screw placement for patients with OPLL were analyzed applying multivariate logistic regression analysis, and the outcomes are shown as odds ratios (ORs) and 95% confidence intervals (CIs). Significance was indicated at the *p* < 0.05 level.

## Results

### Patients population

Forty patients with multilevel cervical OPLL received surgeries with cervical LF and selected for the current study. Twenty-three patients were treated with C2 pedicle screws, and 17 patients were treated with C2 pars screws. The patients’ features are summarized in Table 1. No statistically significant variation in age, sex, type of OPLL, number of smokers or incidence of diabetes mellitus between the two groups.

**Table 1:** Patient Backgrounds

	Pedicle group	Pars group	*p*-value
No. of patients	23	17	
Age (year)	57.04 ± 8.84	58.88 ± 7.75	0.498^1^
Sex (male/female)	15/8	11/6	0.973^2^
Type of OPLL			1.000^3^
Continuous	10	7	
Segmental	1	0	
Mixed	12	10	
Smoker (N)	10	7	0.884^2^
DM (N)	6	3	0.803^4^

*OPLL* Ossification of the posterior longitudinal ligament, *BMI* Body mass index, *DM* Diabetes mellitus

^1^Independent t-test

^2^Chi-square test

^3^Fisher’s Exact test

^4^Continuity Correction test

### Clinical characteristics

The average length of hospitalization (LOH) postoperatively was 5.87 in the pedicle group and 5.18 in the pars group (*p* = 0.055). The pedicle group was related to a longer operation duration and more blood loss than the pars group (operation time: 133.48 ± 26.22 vs 115.29 ± 28.75, *p* = 0.044; blood loss: 457.83 ± 145.45 vs 383.53 ± 116.19, *p* = 0.039). Of all the C2 screws with postoperative CT imaging, none were regarded unacceptable (Type III). In the pedicle group, 42 pedicle screws were considered ideal (Type I), and 4 pedicle screws were considered acceptable (Type II). However, all pars screws were considered ideal (Type I) in the pars group. Following surgery, the idealization and acceptability of C2 screws in the pars group exceeded those in the pedicle group (91.3% vs 100%). Complications included VA injury (pedicle group: 0, pars group: 0), spinal cord injury (pedicle group: 0, pars group: 0), neurologic injury (pedicle group: 0, pars group: 0), CSF leakage (pedicle group: 0, pars group: 0) and screw looseness/failure (pedicle group: 0, pars group: 0) (Table 2).

**Table 2:** Clinical Characteristics

	Pedicle group	Pars group	*p*-value
Operation time (minutes)	133.48 ± 26.22	115.29 ± 28.75	0.044^1^
LOH (days)	5.87 ± 1.06	5.18 ± 1.19	0.055^2^
Blood loss (ml)	457.83 ± 145.45	383.53 ± 116.19	0.039^2^
Screw placement			0.499^3^
Type I Ideal	42	34	
Type II Acceptable	4	0	
Type III Unacceptable	0	0	
% Ideal or Acceptable	91.3%	100%	
No. of complications (N)	1	1	
VA injury	0	0	
Spinal cord injury	0	0	
Neurologic injury	0	0	
CSF leakage	1	1	
Screw looseness/failure	0	0	

*LOH* Length of hospitalization at postoperative, *VA* Vertebral artery, *CSF* Cerebrospinal fluid

^1^Independent t-test

^2^Mann-Whitney U test

^3^Fisher’s Exact test

### Quality of life parameters

Both pedicle and pars groups were associated with significant improvements in the majority of health-related outcomes (Table 3). The mean JOA improved gradually from 10.09 to 13.74 in the pedicle group and from 10.18 to 13.82 in the pars group. The RR was 52.37 ± 24.29% in the pedicle group and 54.98 ± 21.39% in the pars group. The mean VAS score for neck pain decreased from 3.87 to 2.43 in the pedicle group and 3.59 to 2.82 in the pars group. The incidence of axial pain was 30.4% in the pedicle group and 29.4% in the pars group. The mean NDI decreased from 19.17 to 12.22 in the pedicle group and from 20.18 to 11.94 in the pars group. However, no statistically significant difference in any health-related outcomes between the pedicle and pars groups was observed.

**Table 3:** Comparison of Quality of Life Parameters

	Pedicle group	Pars group	*p*-value
**JOA (score)**			
Pre	10.09 ± 1.59	10.18 ± 1.47	0.717^2^
F/U	13.74 ± 1.48	13.82 ± 1.88	0.570^2^
Pre vs F/U	< 0.001^4^	< 0.001^4^	
**RR (%)**	52.37 ± 24.29%	54.98 ± 21.39%	0.558^2^
**RR classification (N)**			0.896^3^
Excellent	3	3	
Good	11	9	
Fair	6	4	
Poor	3	1	
**VAS in neck (score)**			
Pre	3.87 ± 1.18	3.59 ± 0.94	0.671^2^
F/U	2.43 ± 1.27	2.82 ± 1.24	0.340^1^
Pre vs F/U	< 0.001^4^	< 0.001^4^	
**Incidence of axial pain**	30.4%	29.4%	1.000^5^
**NDI (score)**			
Pre	19.17 ± 4.69	20.18 ± 5.51	0.680^2^
F/U	12.22 ± 3.49	11.94 ± 3.94	0.989^2^
Pre vs F/U	< 0.001^4^	< 0.001^4^	

*Pre* Preoperative, *F/U* Follow up, *JOA* Japanese Orthopaedic Association, *RR* Recovery Rate, *VAS* Visual analog scale, *NDI* Neck disability index

^1^Independent t-test

^2^Mann-Whitney U test

^3^Fisher’s Exact test

^4^Wilcoxon Signed Ranks test

^5^Continuity correction test

### Multiple logistic regression

The variables from the univariate analysis that were associated with the pars group were operation duration and blood loss. Various logistic regression analyses demonstrated that operation time and blood loss were both independently related to the pars group (operation time: OR = 0.966, *p* = 0.021; blood loss: OR = 0.993, *p* = 0.046) (Table 4).

**Table 4:** Multiple regression analysis

Variable	OR	*P*	95%CI
Operation time	0.966	0.021*	0.938–0.995
Blood loss	0.993	0.046*	0.986–1.000

*OR* Odds ratio, *CI* Confidence interval

^*^p < 0.05

## Discussions

Surgical options for C2 OPLL involving anterior procedures (anterior cervical corpectomy and fusion) and posterior procedures (laminoplasty, laminectomy and laminectomy with fusion) have been established [16]. However, the optimum method for C2 OPLL is still controversial because of the complex structures around the C2 level. Anterior procedures can provide direct spinal cord decompression and achieve good results, especially when there are few ossified segments and the occupying rate of spinal canal of > 60% [17]. Sun, J., et al. [18] proposed a modified anterior controllable antedisplacement and fusion, which is a “shelter technique” to treat patients with OPLL extending to C2. However, considering the limitations of the sheltered space and the difficulty of intraoperative osteotomy, this surgical technique has not been popularized. In addition, the higher operation level, longer surgery segments, the thickness of the cervical plate, and the stretching or injury of the esophagus during exposure also affect the clinical prognosis [19]. At this moment, posterior approach has shown its advantages, especially when OPLL involves a wide range of segments or with developmental cervical spinal stenosis. It allows adequate indirect decompression through the floating spinal cord, which is safer and easier to obtain good results [20].

There have been many reports on the placement technology of C2 pars screws and C2 pedicle screws, with slightly different starting points and placement trajectories [21–23]. Compared with the C2 pedicle screw, the starting point of the C2 pars screw is closer to the caudal side of the lateral mass, and the placement trajectory is also steeper. Hoh et al. [24] recommended a screw length of 14–16 mm to achieve maximum safety. When we insert the C2 pedicle screw, to avoid damage to the VA, the starting point is often more lateral, and the abduction angle is larger. At the same time, studies have shown that for patients with atlantoaxial screw rod bone graft fusion, the ideal degree of pars screw placement after postoperative CT examination is better than that of pedicle screws [4, 25]. At the same time, a previous review also showed that [26] the risk of VA injury with C2 pedicle screws is approximately 0.3%, while the shorter pars screws have no VA injury. Evidenced by CT, pedicle screws have a higher proportion of screw misalignment than pars screws. This is obviously because the pars screws are shorter and have a safer nail trajectory. However, there are different opinions that although the length of the pars screw is short, it may damage the transverse hole during insertion. Therefore, application of the pars screw does not necessarily reduce the risk of VA injury [27].

In the past 10 years, we have noticed some general trends in our hospital for C2 posterior fixation in patients with OPLL involving the C2 segment. The use of C2 pedicle screws seems to decrease with the appearance of C2 pars screws, indicating that the latter has become the preferred fixation method for patients with challenging VA anatomy. Especially in elderly patients, due to the abnormal and fragile vertebral bone caused by osteoporosis, the use of pedicle screws will have a high risk of VA injury, while short pars screws are usually used when the VA is abnormal or pedicle screw fixation is used when the risk is too high, which indicates that it has become the screw of choice for patients with challenging VA anatomy. Our study found that with the same segmental fixation, the postoperative health-related outcomes of the two groups did not show significant differences, which may be related to the fact that both groups of patients received adequate decompression. However, the operation time and blood loss related to C2 pars screws were significantly less than those of C2 pedicle screws. This may be because compared with a C2 pedicle screw, a C2 pars screw has less muscle dissection on C2 lateral mass and back of lamina and retains the C2 attachment muscle group to a large extent, which reduces partial blood loss. At the same time, the tail direction of C2 pars screws is more medial than C2 pedicle screws, so it is more conducive to the connection of the screw rod system, which saves time in bending and inserting the connecting rod. In addition, O-arm navigation is required for C2 pedicle screw placement, which undoubtedly increases the operation time and repeated adjustment of the screw placement direction also indirectly increases unnecessary blood loss. However, as long as the C2 epidural venous plexus is not damaged, there will be no more blood loss during C2 pedicle screw placement. We think this is an invasion of the muscle, but if it is separated under the periosteum, the muscle will hardly bleed. So the difference in our research may be related to exposure. In addition, for highly skilled surgeons, the implantation of C2 pedicle screws is performed without O-arm navigation and normal fluoroscopy imaging. Therefore, the advantages of pars screw technology in operation time and blood loss may be weakened. According to previous reports, the rate of VA damage and imaging screw dislocation rate of C2 pars screws were both significantly lower than those of C2 pedicle screws. None of the C2 pedicle screws or pars screws had VA, spinal cord, neurologic injury or screw looseness/failure in our study. One patient in both groups showed CSF leakage. The incidence of screw misalignment is also much lower than that reported in previous studies. The high extent of accuracy was attributed to the excellent surgical technique. The ideal and acceptable degree of screw placement in the pedicle group was 91.3%, while the ideal and acceptable degree of screw placement in the pars group was as high as 100%. Although it has not been proven, we believe it is due to the emergence of C2 pars screw technology. Further, the high rate of success also demonstrates that when we have thorough knowledge of pivotal anatomy, all C2 pars screws and C2 pedicle screws can be placed accurately and risk of VA damage or serious clinical dislocation can be avoided. However, biomechanical studies have proven that C2 pedicle screws have greater rigidity and higher load failures than C2 pars screws [28, 29]. The importance of this difference is unclear [30]. If it is necessary to use pedicle screws instead of pars screws, we need to make the delicate trade-off between safety and rigidity, carefully check the preoperative CT, decide which type of screw to use, and consider the habits of surgeons and the actual clinical situation. Therefore, according to our experience, for patients with OPLL involving the C2 segment, we believe that C2 pars screw placement is a secure and effective technique.

However, there are still several weaknesses to be further emphasized and discussed regarding the current research. The first limitation is related to the small-sized retrospective sample. All patients were chosen based on strict inclusion and exclusion criteria. Second, the average follow-up time was too short. A prospective research with a longer follow-up period is necessary to further observe the postoperative clinical results of patients with OPLL. Third, we can only judge that the biomechanical strength of C2 pars screws is sufficient from the previous literature on atlantoaxial internal fixation. Although our study showed that there was no screw looseness or failure in either group within 2 years after the operation, it has not been proven by finite element analysis that patients with OPLL can obtain enough holding force, similar to C2 pedicle screws, after inserting C2 pars screws. A longer follow-up period (e.g., more than 5 years) to observe the screw loosening is very necessary, which is also the focus of future research. Nevertheless, despite these restrictions, our research is valuable in demonstrating the advantages of C2 pars screw placement in patients with OPLL involving the C2 segment.

## Conclusions

In the treatment of patients with OPLL involving the C2 segment, the application of C2 pars screws is an alternative choice, which is not only safer but also reduces the amount of blood loss, shortens the operation time and obtains a more ideal screw placement.

## Data Availability

The datasets generated and analysed during the current study are availabled from the corresponding author on reasonable request.

## Abbreviations

OPLL: Ossification of posterior longitudinal ligament
LF: Laminectomy with fusion
VA: Vertebral artery
CSF: Cerebrospinal fluid
LOH: Length of hospitalization
JOA: Japanese Orthopaedic Association
RR: Recovery Rate
NDI: Neck Disability Index
VAS: Visual Analogue Scale
OR: Odds ratio
CI: Confdence interval

## References

[1] Abiola R, Rubery P, Mesfin A (2016). Ossification of the posterior longitudinal ligament: etiology, diagnosis, and outcomes of nonoperative and operative management. Global Spine J.

[2] Saetia K, Cho D, Lee S, Kim DH, Kim SD (2011). Ossification of the posterior longitudinal ligament: a review. Neurosurg Focus.

[3] Haller JM, Iwanik M, Shen FH (2011). Clinically relevant anatomy of high anterior cervical approach. Spine.

[4] Aryan HE, Newman CB, Nottmeier EW, Acosta FL, Wang VY, Ames CP (2008). Stabilization of the atlantoaxial complex via C-1 lateral mass and C-2 pedicle screw fixation in a multicenter clinical experience in 102 patients: modification of the Harms and Goel techniques. J Neurosurg Spine.

[5] Harms J, Melcher RP (2001). Posterior C1-C2 fusion with polyaxial screw and rod fixation. Spine.

[6] Xiao ZM, Zhan XL, Gong DF, Chen QF, Luo GB, Jiang H (2008). C2 pedicle screw and plate combined with C1 titanium cable fixation for the treatment of atlantoaxial instability not suitable for placement of C1 screw. J Spinal Disord Tech.

[7] Xie N, Ni B, Chen DY, Ye XJ, Xiao JR, Yuan W (2008). Combined anterior C2,3 reduction and fusion with posterior compressive C2 pedicle screw fixation for the treatment of unstable Hangman's fractures: 16 cases review. Zhonghua Wai Ke Za Zhi.

[8] Goel A, Laheri V (1994). Plate and screw fixation for atlanto-axial subluxation. Acta Neurochir.

[9] Gupta S, Goel A (2000). Quantitative anatomy of the lateral masses of the atlas and axis vertebrae. Neurol India.

[10] Gorek J, Acaroglu E, Berven S, Yousef A, Puttlitz CM (2005). Constructs incorporating intralaminar C2 screws provide rigid stability for atlantoaxial fixation. Spine.

[11] Lapsiwala SB, Anderson PA, Oza A, Resnick DK (2006). Biomechanical comparison of four C1 to C2 rigid fixative techniques: anterior transarticular, posterior transarticular, C1 to C2 pedicle, and C1 to C2 intralaminar screws. Neurosurgery.

[12] Parker SL, McGirt MJ, Garces-Ambrossi GL, Mehta VA, Sciubba DM, Witham TF, Gokaslan ZL, Wolinksy JP (2009). Translaminar versus pedicle screw fixation of C2: comparison of surgical morbidity and accuracy of 313 consecutive screws. Neurosurgery.

[13] Sciubba DM, Noggle JC, Vellimana AK, Conway JE, Kretzer RM, Long DM, Garonzik IM (2008). Laminar screw fixation of the axis. J Neurosurg Spine.

[14] Wang MY (2007). Cervical crossing laminar screws: early clinical results and complications. Neurosurgery.

[15] Upendra BN, Meena D, Chowdhury B, Ahmad A, Jayaswal A (2008). Outcome-based classification for assessment of thoracic pedicular screw placement. Spine.

[16] Lee SE, Jahng TA, Kim HJ (2016). Surgical outcomes of the ossification of the posterior longitudinal ligament according to the involvement of the C2 segment. World Neurosurg.

[17] Fujimori T, Iwasaki M, Okuda S, Takenaka S, Kashii M, Kaito T, Yoshikawa H (2014). Long-term results of cervical myelopathy due to ossification of the posterior longitudinal ligament with an occupying ratio of 60% or more. Spine.

[18] Sun J, Sun K, Wang S, Yang H, Wang Y, Xu X, Shi J (2019). "shelter technique" in the treatment of ossification of the posterior longitudinal ligament involving the C2 segment. World Neurosurg.

[19] Kimura H, Shikata J, Odate S, Soeda T (2014). Anterior corpectomy and fusion to C2 for cervical myelopathy: clinical results and complications. Eur Spine J.

[20] Iwasaki M, Kawaguchi Y, Kimura T, Yonenobu K (2002). Long-term results of expansive laminoplasty for ossification of the posterior longitudinal ligament of the cervical spine: more than 10 years follow up. J Neurosurg.

[21] Chen JF, Wu CT, Lee SC, Lee ST (2005). Posterior atlantoaxial transpedicular screw and plate fixation. Technical note. J Neurosurg Spine.

[22] Chun HJ, Bak KH (2011). Targeting a safe entry point for c2 pedicle screw fixation in patients with atlantoaxial instability. J Korean Neurosurg Soc.

[23] Payer M, Luzi M, Tessitore E (2009). Posterior atlanto-axial fixation with polyaxial C1 lateral mass screws and C2 pars screws. Acta Neurochir.

[24] Hoh DJ, Liu CY, Wang MY (2010). A radiographic computed tomography-based study to determine the ideal entry point, trajectory, and length for safe fixation using C-2 pars interarticularis screws. J Neurosurg Spine.

[25] Ondra SL, Marzouk S, Ganju A, Morrison T, Koski T (2006). Safety and efficacy of C2 pedicle screws placed with anatomic and lateral C-arm guidance. Spine.

[26] Elliott RE, Tanweer O, Boah A, Smith ML, Frempong-Boadu A (2012). Comparison of safety and stability of C-2 pars and pedicle screws for atlantoaxial fusion: meta-analysis and review of the literature. J Neurosurg Spine.

[27] Helgeson MD, Lehman RA, Dmitriev AE, Kang DG, Sasso RC, Tannoury C, Riew KD (2011). Accuracy of the freehand technique for 3 fixation methods in the C-2 vertebrae. Neurosurg Focus.

[28] Du JY, Aichmair A, Kueper J, Wright T, Lebl DR (2015). Biomechanical analysis of screw constructs for atlantoaxial fixation in cadavers: a systematic review and meta-analysis. J Neurosurg Spine.

[29] Dmitriev AE, Lehman RA, Helgeson MD, Sasso RC, Kuhns C, Riew DK (2009). Acute and long-term stability of atlantoaxial fixation methods: a biomechanical comparison of pars, pedicle, and intralaminar fixation in an intact and odontoid fracture model. Spine.

[30] Mummaneni PV, Lu DC, Dhall SS, Mummaneni VP, Chou D (2010). C1 lateral mass fixation: a comparison of constructs. Neurosurgery.

